# Effect of *Bacillus coagulans* BC99 supplementation on body weight and gut microbiota in overweight and obese individual: a randomized, double-blind, placebo-controlled study

**DOI:** 10.3389/fnut.2025.1542145

**Published:** 2025-05-09

**Authors:** Rui Wang, Guoming Zhang, Xiaoya Wang, Zefeng Xing, Zhen Li, Lixiang Li

**Affiliations:** ^1^Department of Gastroenterology, Qilu Hospital of Shandong University, Jinan, Shandong, China; ^2^Shandong Provincial Clinical Research Center for Digestive Disease, Jinan, Shandong, China; ^3^Laboratory of Translational Gastroenterology, Qilu Hospital of Shandong University, Jinan, Shandong, China; ^4^Robot Engineering Laboratory for Precise Diagnosis and Therapy of GI Tumor, Qilu Hospital of Shandong University, Jinan, Shandong, China

**Keywords:** *B. coagulans*, gut microbiota, overweight, obese, probiotics

## Abstract

**Introduction:**

Probiotic supplementation is a safe and effective way to reduce overweight and obesity by regulating the gut microbiota. The *Bacillus coagulans* strain BC99 is derived from humans and has various probiotic-related, acid resistance, bile salt resistance, and adhesion-related domains in the genome. This study aimed to assess the effects of BC99 on the gut microbiota, body weight and lipid profiles of overweight and obese individuals.

**Methods:**

A total of 66 adult individuals were randomly assigned to a probiotic group (supplemented with 5 × 10^9^ colony-forming units of BC99 per day along with 3 g of maltodextrin) and placebo group (supplemented with 3 g of maltodextrin daily) in a 1:1 ratio.

**Results:**

After 8 weeks of oral administration, BC99 intervention significantly decreased the body weight of overweight individual (*P* < 0.01). Weight loss was significantly greater in the probiotic group than in the placebo group (*P* < 0.05). No significant differences were observed in lipid profiles between two groups. The microbiota analysis revealed that BC99 intervention significantly improved the β-diversity at week 4. The genus *Parabacteroides* was negatively correlated with body weight and was found to be enriched in the BC99 group.

**Discussion:**

These findings suggest that *B. coagulans* strain BC99 could be a beneficial candidate for modulating the gut microbiota and improve body weight management for overweight individuals.

**Clinical trial registration:**

ClinicalTrials.gov, identifier NCT06077383.

## 1 Introduction

Obesity has become a significant global health concern owing to the impact of an unhealthy lifestyle on individuals' wellbeing, driven by rapid economic growth in the recent decades (1, 2). Overweight and obesity are ranked as the fifth leading causes of mortality worldwide (3). Obesity-related deaths are primarily attributed to chronic metabolic diseases such as type 2 diabetes, insulin resistance, non-alcoholic fatty liver disease, hyperlipidemia, and cardiovascular diseases (3). China is one of the most populous countries worldwide. The expected increase in the prevalence of obesity and overweight among Chinese adults is predicted to reach 70.5% by 2030, which will affect approximately 810 million individuals and remains an urgent problem (4). Therefore, safe and effective methods to combat obesity are of great significance and need to be found.

The gut microbiota plays an essential role in human health (5) and is modulated by multiple factors in the human body, including diet, genetics, antibiotics, probiotics, and physical fitness (6, 7). Obesity has been associated with gut microbiota dysbiosis, including changes in the diversity and community of the gut microbiota, and the presence and abundance of particular bacteria (8–10). Probiotics can provide anti-obesity effects by inhibiting the differentiation of preadipocytes, maintaining the balance of T helper (Th)1/Th2 cytokines, changing the composition of the intestinal microbiota, reducing the lipid level and regulating energy metabolism. Accumulating evidence suggests that probiotic supplementation is a safe and effective method to reduce overweight and obesity by modulating the gut microbiota (11).

Probiotics contain various microorganisms, including strains of genus *Lactobacillus, Bifidobacterium, Bacillus, Pediococcus, Clostridium, Saccharomyces etc*. Lactic acid bacteria are currently the most common microbial type used as probiotics and have a variety of health benefits, such as regulation of the immune system and improvement of intestinal function. *Bacillus coagulans* (also named *Weizmannia coagulans*) BC99 is a high-performance lactic acid-producing bacterium with high viability and stability, and it provides numerous health benefits to the host, including intestinal flora balance regulation, intestinal motility restoration, inhibition of pathogen growth and adhesion, and short chain fatty acids production (12, 13). The clinical trial has found that *B. coagulans* strain BC99 can improve constipation symptoms and regulate intestinal flora in adults with chronic constipation. No obvious adverse reactions were observed in all patients, confirming the safety of *B. coagulans* strain BC99 (14).

Complete-genome sequencing of BC99 revealed that its genome contains probiotic-related, acid resistance, bile salt resistance, and adhesion-related domains (15). This suggests that BC99 may have the ability to possess the potential of inhibiting fat synthesis. Previous studies have revealed that the BC99 supplementation may benefit individuals with obesity by reducing visceral fat mass (16, 17). However, few studies have investigated the effects of BC99 supplementation on obesity management. In this study, we aimed to assess the effects of BC99 on the gut microbiota, body weight and lipid profiles of overweight and obese individuals.

## 2 Materials and methods

### 2.1 Participants

A total of 85 overweight and obese subjects were recruited at Qilu Hospital of Shandong University between September 2023 and August 2024 under the following inclusion criteria: (1) being between the ages of 18 and 65, and (2) having a body mass index (BMI) above 24 30 kg/m^2^. The Working Group on Obesity in China recommended BMI cutoffs of 24.0 and 28.0 to define overweight and obesity, respectively (18) (3) no reduction in food intake and ability to accept dietary interventions; (4) commitment to complying with research requirements and procedures; and (5) having signed informed consent forms. The exclusion criteria were as follows: (1) use of antibiotics, probiotics or any other drugs which could influence the gut microbiota 3 months prior to the study; (2) cessation of provision of fecal samples for testing or using other drugs midway through the trial; (3) obesity caused by drugs; (4) being pregnant or lactating; (5) neuroendocrine system diseases; (6) previous history of gastrointestinal diseases (such as irritable bowel syndrome, celiac disease, inflammatory bowel disease or other diseases) and surgical history; (7) hereditary obesity, metabolic obesity, endocrine obesity, any disease affecting liver fibrosis or steatosis; (8) change in diet habits during the study period; (9) severe diseases, such as diseases affecting cardiovascular, cerebrovascular, as well as hematopoietic systems; (10) being in a condition that does not qualify for study participation, as judged by the researcher.

### 2.2 Study design and intervention

This single-center, randomized, double-blind, placebo-controlled trial was approved by the Medical Ethics Committee of the Qilu Hospital of Shandong University (approval number: KYLL-202307-008). All the participants were fully informed of the possible benefits and potential risks of participating in this trial, and voluntarily signed a written informed consent form. The trial is registered at ClinicalTrials.gov (accession ID: NCT06077383).

Eligible patients were randomly assigned to a probiotic or placebo group in a 1:1 ratio. The randomization was carried out via a random number sequence obtained using SAS^®^ 9.4 (SAS Institute Inc., Cary, NC, USA). The participants and clinical researchers were blinded to the randomization and the products used in the study.

The probiotic group was supplemented with the probiotic (*B. coagulans* BC99, 5 × 10^9^ CFU/day and maltodextrin, 3 g/day), and the placebo group was supplemented with a placebo (3 g maltodextrin) once daily for 8 weeks. To ensure double blinding, the probiotic and placebo were prepared by WeCare Probiotics Co., Ltd., Jiangsu, P.R. China, using the same packet. The participants were requested to maintain their diet and exercise and were instructed not to consume any other probiotics or dietary supplements containing probiotics.

Eligible participants were identified during the screening period (day 0) and their baseline demographic data were collected. Subsequently, the participants received probiotic or placebo treatment (day 1 to day 56, week 8).

### 2.3 Outcomes

The primary outcomes were changes in gut microbes at baseline, week 4 and week 8. The secondary outcomes were changes in body weight and blood lipid profiles. Body weight was recorded on day 0, week 4, and week 8. Total cholesterol (TC), triglyceride (TG), low-density lipoprotein cholesterol (LDL-C), and high-density lipoprotein cholesterol (HDL-C) levels were measured using a Roche Cobas 8,000 modular analyzer system (Roche Diagnostics, IN, USA).

### 2.4 Fecal samples collection

The fecal samples of the participants were collected before the start of treatment (week 0), on the day 28 (week 4) and on day 56 (week 8) in disposable sterile fecal collection tubes and immediately frozen at −80°C.

### 2.5 DNA extraction, polymerase chain reaction (PCR) amplification, and sequencing

The genomic DNA from the stool samples was extracted using the E.Z.N.A.^®^ soil DNA Kit (Omega Bio-tek, Norcross, GA, U.S.) and measured via 1.0% agarose gel electrophoresis as well as a NanoDrop^®^ ND-2000 spectrophotometer (Thermo Scientific Inc., USA) to evaluate the quality and concentration. The V3-V4 region of the bacterial 16S rRNA gene were amplified using the primers 338F (5′-ACTCCTACGGGAGGCAGCAG-3′) and 806R (5′-GGACTACHVGGGTWTCTAAT-3′) (19). All samples were amplified in triplicates. The PCR products were extracted using 2% agarose gel and purified using the AxyPrep DNA Gel Extraction Kit (Axygen Biosciences, Union City, CA, USA). The PCR products obtained were further quantified using Quantus™ Fluorometer (Promega, USA) and then sequenced using the Illumina MiSeq PE300 platform (Illumina, San Diego, USA). This process was performed by Majorbio Bio-Pharm Technology Co. Ltd. (Shanghai, China).

### 2.6 Data processing and microbiota analysis

After demultiplexing, the resulting sequences were quality filtered using fastp (0.19.6) (20) and merged using FLASH (v1. 2.11) (21). The high-quality sequences were denoised using the DADA2 (22) plugin in the Qiime2 (version 2020.2) pipeline (23) based on the recommended parameters to obtain amplicon sequence variants (ASVs). The taxonomic assignment of ASVs was performed using the naive Bayes consensus taxonomy classifier implemented in Qiime2 based on the SILVA 16S rRNA database (v138).

Bioinformatics analysis was conducted using the Majorbio Cloud platform (https://cloud.majorbio.com). Alpha diversity indices, including Chao1 richness, Shannon index, Ace index, and Simpson index, were calculated using Mothur v1.30.1, based on the ASV information (24). The similarity among the microbial communities in different fecal samples was assessed using principal coordinate analysis using the Vegan v2.5-3 package based on Bray-Curtis dissimilarity. Additionally, linear discriminant analysis (LDA) effect size (LEfSe) was employed to identify significantly abundant taxa of microbes in the different groups or in the samples collected from the same group at different times, with an LDA score>2 and *P* < 0.05 (25).

### 2.7 Statistical analysis

Statistical analyses were performed using the SPSS software (IBM, Armonk, NY, USA). Continuous data were presented as mean ± standard deviation. The Shapiro–Wilk test was used to ensure a normal distribution of body weight and serum index. The pre- and post-treatment body weights and serum indices were compared within each group using a paired-sample *t*-test. An independent samples *t*-test was used to evaluate the differences between the two groups at each time point. *P* < 0.05 were considered significant.

## 3 Results

### 3.1 Baseline characteristics and enrollment of participants

**Figure 1:**
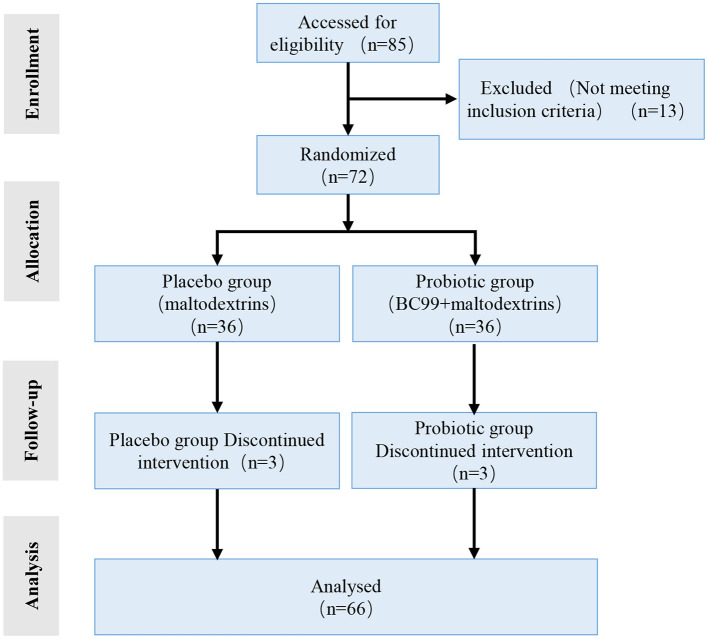
Flow diagram showing the study design.

A total of 66 overweight and obese individuals who met the inclusion and exclusion criteria were enrolled. They were randomly assigned in a 1:1 ratio to the probiotic (*n* = 33) or placebo groups (*n* = 33). A flow diagram of the trial is shown in Figure 1. The average age of the participants and the proportion of male participants, respectively, were 36.21 ± 10.39 years and 51.5% (17/33) in the placebo group and 33.03 ± 9.18 years and 45.5% (15/33) in the probiotic group. There were no significant differences in age, sex, or BMI between the two groups (*P* > 0.05) (Table 1). Among the participants in each group, 20 were overweight, and 13 were obese.

**Table 1:** Participant characteristics by group.

**Characteristic**		**Placebo group (*n* = 33)**	**BC99 group (*n* = 33)**	***P*-Value**
Sex	Men	17	15	0.80
	Women	16	18	
Age, years		36.21 ± 10.39	33.03 ± 9.18	0.13
Height, cm		169.7 ± 8.16	170.1 ± 9.45	0.86
Weight, kg		80.80 ± 12.47	82.53 ± 12.67	0.58
Body mass index, kg/m^2^		27.98 ± 3.20	28.46 ± 3.22	0.55

### 3.2 Effects of BC99 supplementation on body weight and lipid profiles of the participants

After 4 weeks of probiotic intervention, we compared the effects of BC99 supplementation on body weight between the probiotic and placebo groups. As shown in Table 2, both BC99 (*P* < 0.0001) and placebo (*P* < 0.01) significantly reduced the body weight of the participants; no significant difference was observed between the two groups. The results after 8 weeks of probiotic intervention remained consistent with those observed at 4 weeks.

**Table 2:** The weight change from baseline according to group.

**Group**	**Treatment**	**Baseline**	**4 week**	**8 week**	***P*^##^-Value (Baseline-4 week)**	***P*^##^-Value (Baseline-8 week)**
Overweight+ Obese	BC99 (*n* = 33) Placebo (*n* = 33)	82.53 ± 12.67 80.80 ± 12.47	81.38 ± 12.76 79.36 ± 11.31	80.39 ± 12.74 78.51 ± 10.89	<0.0001 <0.01	<0.0001 <0.01
	*P*^#^-Value	0.58	0.50	0.52		
Overweight	BC99 (*n* = 20) Placebo (*n* = 20)	77.95 ± 10.90 74.45 ± 7.95	76.93 ± 10.80 73.77 ± 7.44	76.07 ± 10.73 73.54 ± 7.76	<0.01 0.05	<0.01 0.05
	*P*^#^-Value	0.25	0.29	0.40		
Obese	BC99 (*n* = 13) Placebo (*n* = 13)	89.58 ± 12.30 90.58 ± 11.99	88.23 ± 12.87 87.96 ± 11.02	87.04 ± 13.09 86.16 ± 10.78	<0.01 < 0.05	<0.01 0.04
	*P*^#^-Value	0.84	0.95	0.85		

Values are represented as mean ± SD.

P ^#^-Value, P-values between the probiotic group and the placebo group at the same time point; P ^##^-Value, intra-group P-values in week 0, week 4 and week 8.

We compared the body weights of the overweight and obese subgroups across the two groups. The body weight of the overweight subgroup decreased significantly after the probiotic BC99 intervention (*P* < 0.01) but did not change significantly after being supplemented with placebo (*P* = 0.05). Weight loss was significantly greater in the probiotic group than in the placebo group (Supplementary Figure S1, *P* < 0.05). The body weight of the obese subgroup in the two groups decreased significantly after the intervention (*P* < 0.05); there was no significant difference between the two groups.

We also compared the lipid profiles between the probiotic and placebo groups after the intervention (Table 3). Neither the probiotic BC99 nor placebo had a significant effect on TC, TG, HDL-C, and LDL-C levels at weeks 4 and 8.

**Table 3:** The change of weight and lipid profile at 4 weeks from baseline according to group.

**Parameter**	**Treatment**	**Baseline**	**4 week**	**8 week**	***P*^##^-Value (Baseline-4 week)**	***P*^##^-Value (Baseline-8 week)**
TC	BC99 Placebo	4.75 ± 1.03 4.83 ± 0.95	4.77 ± 1.03 4.80 ± 0.98	4.88 ± 1.03 4.79 ± 1.00	0.83 0.74	0.30 0.78
	*P*^#^-Value	0.74	0.90	0.71		
TG	BC99 Placebo	1.47 ± 0.80 1.45 ± 0.71	1.49 ± 1.19 1.38 ± 0.76	1.46 ± 0.57 1.39 ± 0.78	0.30 0.79	1.0 0.48
	*P*^#^-Value	0.97	0.84	0.29		
HDL-C	BC99 Placebo	1.24 ± 0.26 1.30 ± 0.30	1.27 ± 0.0.26 1.31 ± 0.27	1.26 ± 0.25 1.34 ± 0.32	0.22 0.73	0.31 0.15
	*P*^#^Value	0.36	0.54	0.31		
LDL-C	BC99 Placebo	2.92 ± 0.81 3.00 ± 0.85	2.96 ± 0.83 3.04 ± 0.83	3.03 ± 0.97 2.88 ± 0.88	0.70 0.62	0.39 0.39
	*P*^#^-Value	0.70	0.69	0.53		

TC, Total cholesterol; TG, triglyceride; LDL-C, low-density lipoprotein cholesterol; HDL-C, high-density lipoprotein cholesterol.

P^#^-Value, P-values between the probiotic group and the placebo group at the same time point; P^##^-Value, intra-group P-values in week 0, week 4 and week 8.

### 3.3 Changes in diversity of the gut microbiota during the probiotic intervention

**Figure 2:**
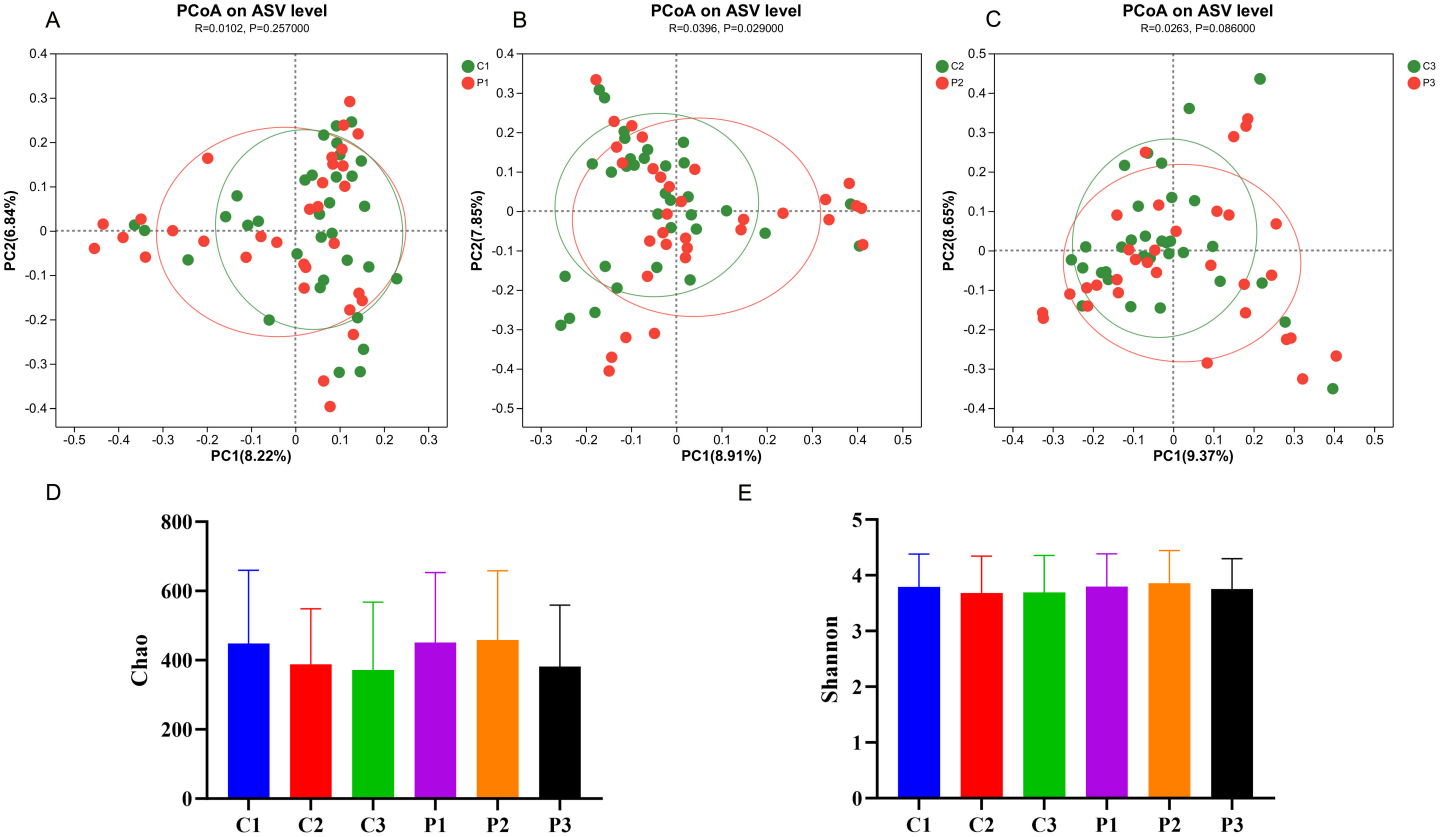
The diversity of the gut microbiota changed during probiotic intervention. **(A–C)** Principal coordinate analysis (PCoA) between the probiotic group and the placebo group at the same time point. **(D)** The Chao index of the microbial diversity in the six groups. **(E)** The Shannon index of the microbial diversity in the six groups. P, probiotic group; C, placebo group; P1/C1, pretreatment; P2/C2, 4 weeks of probiotic intervention; P3/C3, 8 weeks of the probiotic intervention.

16S rRNA gene sequencing of feces from the participans was performed to explore changes in the gut microbiota. A total of 195 samples from all participants were successfully sequenced except from one participant in the placebo group. In total, 12,036,857 raw reads were obtained using high-throughput sequencing, and the number of raw reads per sample ranged from 40,477 to 87,270. To minimize the effects of sequencing depth on microbiota analysis, the sequences from each sample were rarefied to 24,264, with an average Good's coverage above 99.1%. The diversity of the microbiota is shown in Figure 2. The β-diversity of the BC99 and placebo groups were similar before the intervention (*R* = 0.01, *P* = 0.26) (Figure 2A). After 4 weeks of intervention, the β-diversity of the BC99 group was significantly different from that of the placebo group (*R* = 0.04, *P* = 0.03). However, this difference disappeared after 8 weeks (*R* = 0.03, *P* = 0.09). Moreover, the Shannon indices of the microbial communities in the two groups did not change significantly during the intervention. The chao index of the placebo group decreased during the intervention, from 448 ± 211.4 to 387.8 ± 160.3 (*P* = 0.06) at week 4 and then to 371.6 ± 196.1 (*P* = 0.05) at week 8, without significant differences. In contrast, the probiotic group did not show a decrease in chao index at week 4 and only showed a decrease at week 8 (from 451.0 ± 202.3 to 381.4 ± 177.6). Moreover, no significant difference was found in the chao index between the two groups at the same time points.

### 3.4 Changes in the microbial community composition during the probiotic intervention

**Figure 3:**
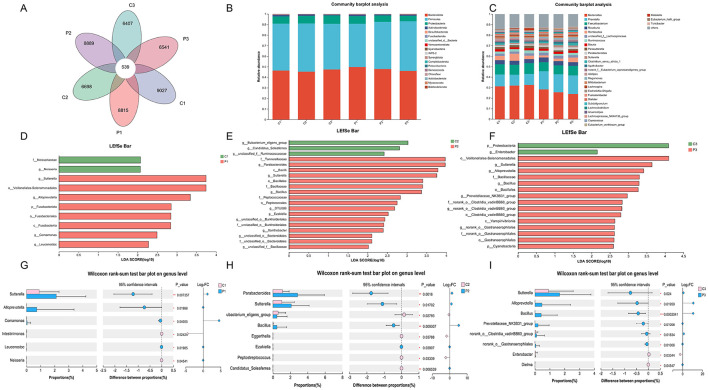
Changes in microbial community composition during probiotic intervention. **(A)** The Venn plot analysis of the six groups. **(B, C)** The microbial relative abundance of the six groups at the phylum and genus levels. **(D–F)** LEfSe analysis of gut microbiota between the probiotic group and the placebo group at the same time point. **(G–I)** Differences in gut microbiota (genus level) between the probiotic group and the placebo group at the same time point. P, probiotic group; C, placebo group; P1/C1, pretreatment; P2/C2, 4 weeks of probiotic intervention; P3/C3, 8 weeks of the probiotic intervention; LDA, linear discriminant analysis.

We then analyzed the microbial community composition during the probiotic intervention and the results are shown in Figure 3. Venn plot analysis based on the ASV showed that the common ASV of each group at different time points is only 539, while the unique ASV of each group exeeeds 6,000 (Figure 3A). The unique ASV of the placebo group at week 4 and week 8 as well as that of the BC99 group, decreased. At the level of phylum, the dominant microbes that accounted for more than 1% of both groups were Bacteroidota, Firmicutes, Proteobacteria and Actinobacteriota (Figure 3B). At the level of genus, the top 10 genera in the groups includeds *Bacteroides, Prevotella, Faecalibacterium, Roseburia, Romboutsia*, unclassified_f__*Lachnospiraceae, Ruminococcus, Blautia, Parasutterella*, and *Parabacteroides* (Figure 3C).

We further analyzed the differences in microbial communities among the different groups at the same time points using LEfSe (Figures 3D–F). The genera of *Parabacteroides, Sutterella, Bacillus* and *Ezakiellla* were enriched in the BC99 group whereas the genera of *Eubacterium*_glilgens_group, Candidatus_Soleaferrea and unclassified_f__*Ruminococcus* were enriched in the placebo group at week 4 (Figures 3G–I). At week 8, the phylum Proteobacteria and genus *Enterobacter* enriched in the placebo group, whereas the genera of *Sutterella, Alloprevotella, and Prevotellaceae_*NK3B31*_*group enriched in the BC99 group. In addition, maltodextrin supplementation significantly enriched *Veillonella, Lactobacillus, Alloprevotella* and *Bacillus* and decrease Collinsella, *Allorhizobium-Neorhizobium-Pararhizobium-Rhizobium, Stenotrophomonas* etc (Supplementary Figure S2).

### 3.5 Correlation of microbiota with body weight and lipid profiles

**Figure 4:**
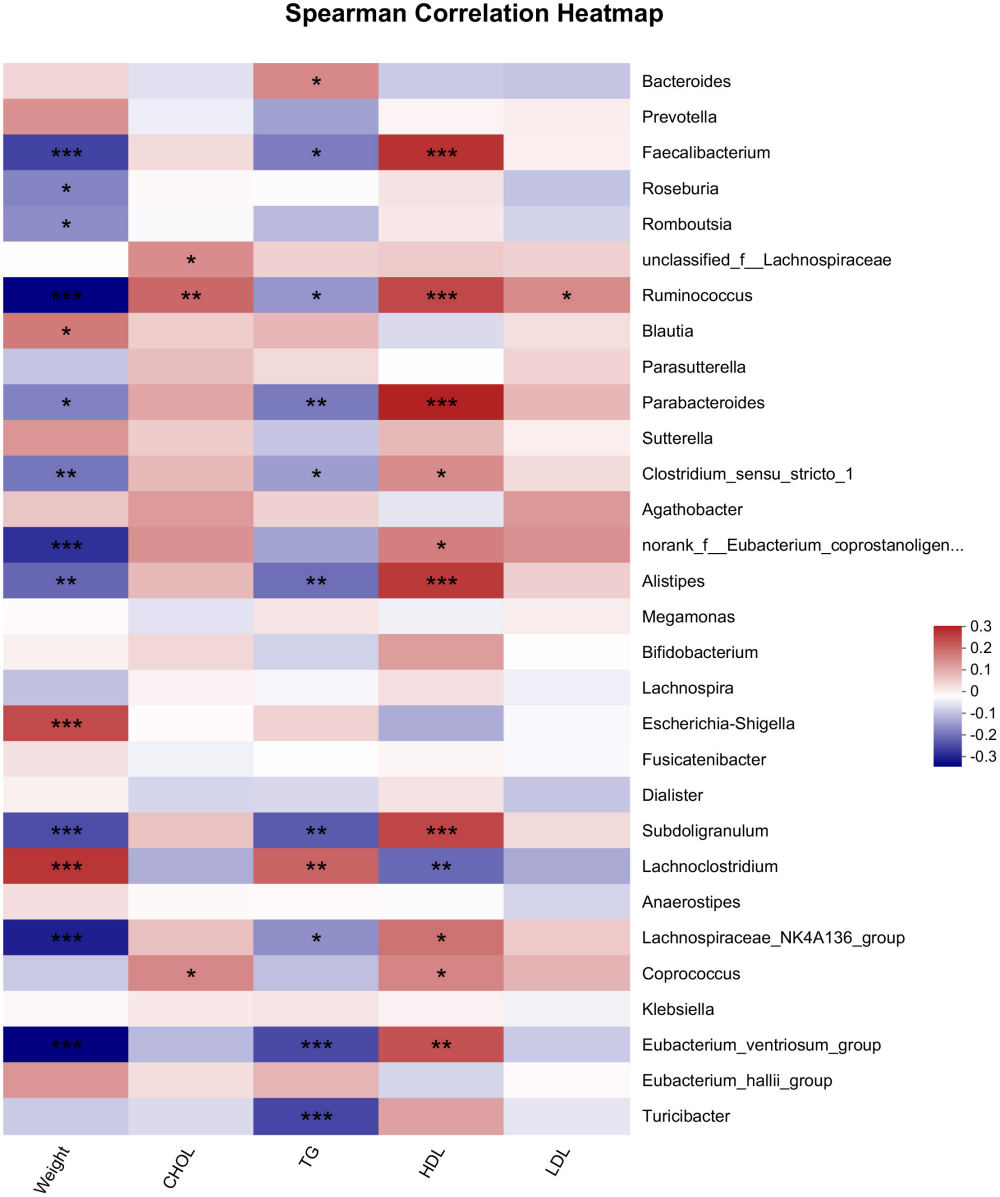
Spearman analysis of microbiota and lipids indicators. ^*^ < 0.05, ^**^ < 0.01, ^***^ < 0.001.

Microbiota is related to body weight and lipid profiles (26, 27). In this study, we used the Spearman analysis to analyze the relationship of microbiota with body weight and lipid profiles (Figure 4). The results illustrated that the genera of *Ruminococcus, Faecalibacterium*, Eubacterium_ventriosum_group, and *Parabacteroides* etc. were significantly negatively related to weight. In contrast, the genera of *Escherichia-Shigella, Lachnoclostrium* and *Blautia* were positively correlated with body weight. Most bacteria related to TG exhibited a similar trend of correlation with TG as well as with body weight. The bacteria significantly associated with HDL were similar to those associated with body weight, whereas the trends for the correlation of these bacteria with HDL-C and body weight were opposite, Only one genus *Ruminococcus* was positively associated with LDL, and three genera including *Ruminococcus, Coprococcus and unclassified_f_Lachnospiraceae*, were positively associated with TC.

## 4 Discussion

The gut microbiota has been increasingly associated with the development of obesity and is becoming a target for new anti-obesity therapies (28–30). A systematic review found that probiotic supplementation is beneficial for managing metabolic indicators (31). B. coagulans is a lactic acid producer with high viability and stability and it provides numerous health benefits for the host (12, 13). However, studies estimating the effects of *B. coagulans* supplementation on obesity management are limited (16, 17). Cho et al. found that an ingredient mixed with six -grain types fermented by *B. coagulans* resulted in a significant reduction in visceral adipose tissue, total fat mass, body weight, and waist circumference (17). Another clinical trial focusing on overweight or obese children and adolescents found that intervention with *B. coagulans* SC-208, *B. indicus* HU36, and fructooligosaccharides significantly decreased the waist-to-height ratio (32). In morbidly obese patients undergoing laparoscopic sleeve gastrectomy, the administration of *B. coagulans* and galactomannans significantly decreased TG, LDL-C, and Aspartate aminotransferase (AST) levels and resulted in significant weight loss (16). Our study found that both BC99 and placebo supplementation significantly decreased the body weight of overweight and obese adults. However, only the BC99 intervention significantly decreased body weight in overweight participants (*P* < 0.01). Further, weight loss was significantly higher in the overweight participants in the probiotic group than in those in the placebo group (*P* < 0.05). This indicates that BC99 supplementation is more suitable for overweight adults.

Many studies have found that the gut microbiota community of overweight individuals could be changed by probiotic intervention, such as *Lactobacillus plantarum, L. acidophilus* and *Bifidobacterium lactis* (11, 33, 34). In an obese mouse model, *B. coagulans* T4 administration successfully mitigated obesity and the related metabolic disorders. *B. coagulans* T4 intervention partly recovered the high-fat diet (HFD)-induced change of gut microbiota, including reduction of the Firmicutes/Bacteriodetes ratio and increase in the number of *Lactobacillus* and *Faecalibacterium* (35). *B. coagulans* BC69 also decreased body weight gain and improved the gut microbiome of high-sugar-high-fat diet-fed mice (36). The addition of *B. coagulans* BC30 can affect glycemia- and lipid-related markers and resistin levels in obese or hyperglycemic individuals (37). These results suggest that *B. coagulans* may play a role in body weight reduction by regulating the gut microbiota in mice. Although *B. coagulans* has demonstrated anti-obesity activity in clinical trials, there is currently no research on its ability to modify the gut microbiota in overweight and obese individuals. In this study, BC99 intervention significantly changed the β-diversity of the gut microbiota at week 4. The genus *Bacillus* was enriched in the probiotic group at weeks 4 and 8, indicating that it adapted well to the intestinal environment. In addition, the genus *Parabacteroides*, which was negatively correlated with body weight, was enriched in the probiotic group. Wang et al. reported that *Parabacteroides* has a significantly negative relationship with BMI. *Parabacteroides distasonis* decreased weight gain in leptin-deficient and HFD-fed mice. The application of *Parabacteroides distasonis* can significantly alter the bile acid profile, increase lithocholic acid and ursodeoxycholic acid, and increase the level of succinate in the intestine, which in turn reduce hyperlipidemia via the activation of the farnesoid X receptor pathway to repair intestinal barrier integrity (38). This indicates that the BC99 may reduce body weight by modulating the gut microbiota.

Maltodextrin is produced from starch using hydrolysis, purification, and spray-drying methods. As a low-sweet food additive, polysaccharides have been used as a placebo to investigate the effects of various interventions, particularly prebiotics (39), probiotics (40), and numerous dietary supplements (41). Almutairi et al. have reported that orally consumed maltodextrin often affects human physiology, including body weight, frailty index, and suprailiac skinfold thickness etc. (42). It can also induce alterations in taxon profiles, such as an increase in *Lactobacillus* (43) and *Lactobacillus-Enterococcus* (44). In this study, we also found that both 4- and 8-week maltodextrin interventions significantly reduced the body weight of overweight and obese participants (Table 2; *P* < 0.01). In addition, we also found that intervention with maltodextrin can reduce the Chao index of the gut microbiota, indicating a decrease in the richness of the gut microbiota. However, the difference was not significant (Figure 2D). In terms of microbial composition, maltodextrin significantly increased *Veillonella, Lactobacillus, Alloprevotella*, and Bacillus and decreased *Collinsella, Allorhizobium*-*Neorhizobium*-*Pararhizobium*-*Rhizobium, Stenotrophomonas* etc. (Supplementary Figure S2). These results help to elucidate the *in vivo* functions of maltodextrin.

This study has some limitations. First, the indicators were recorded only until week 8. Follow-up after discontinuing probiotics might help understand the long-term effects of probiotic application on body weight and the gut microbiota. Secondly, it is impossible to perform a species-level analysis of the gut microbiota based on 16S rRNA sequencing. The identification and quantitative analysis of BC99 and other endogenous Bacillus strains in the gut microbiota were not performed. Third, the direct correlation between BC99 and the modulation of host metabolism remains unclear. Further studies on BC99 and *Parabacteroides* are needed to comprehensively clarify the underlying mechanism of the anti-obesity effect of BC99. Additionally, maltodextrin appears to affect the gut microbiota. Although many studies have used maltodextrin as a placebo, its effects on the gut microbiota require further evaluation.

## 5 Conclusion

BC99 intervention for 8 weeks significantly decreases the body weight and increases weight loss in overweight individuals. It persists in the gut microbiota until week 8 and can increase the quantity of the anti-obesity microbe, *Parabacteroides*. Additionally, it can significantly alter the diversity of the gut microbiota. These findings suggest that BC99 intervention could be a beneficial method to improve body weight by modulating the gut microbiota for overweight individuals.

## Data Availability

The raw data supporting the conclusions of this article will be made available by the authors, without undue reservation.
