# Supplementary material for: Effect of Bacillus coagulans BC99 supplementation on body weight and gut microbiota in overweight and obese individual: a randomized, double-blind, placebo-controlled study
Source: Front Nutr. 2025 May 9;12:1542145. doi: 10.3389/fnut.2025.1542145 (PMC12100662; doi:10.3389/fnut.2025.1542145)
Supplement: Supplementary file 1 [file Data_Sheet_1.docx]

**Supplementary materials for**

**Effect of *Bacillus coagulans* BC99 Supplementation on body weight and gut microbiota in** **overweight and obese individual: a randomized, double-blind, placebo-controlled study**

Rui Wang^1,2,3,4†^, Guoming Zhang^1,2,3,4†^, Xiaoya Wang^1,2,3,4^, Zefeng Xing^1,2,3,4^, Zhen Li^1,2,3,4*^, Lixiang Li^1,2,3,4*^

^1^Department of Gastroenterology, Qilu Hospital of Shandong University, Jinan, Shandong, China

^2^Shandong Provincial Clinical Research Center for Digestive Disease, Shandong, China

^3^Laboratory of Translational Gastroenterology, Qilu Hospital of Shandong University, Jinan, Shandong, China

^4^Robot engineering laboratory for precise diagnosis and therapy of GI tumor, Qilu Hospital of Shandong University, Jinan, Shandong, China

* Correspondence author:

Zhen Li, Address: Department of Gastroenterology, Qilu Hospital of Shandong University, 107 Wenhuaxi Road, Jinan, Shandong Province, China, 250012, Email: qilulizhen@sdu.edu.cn

Lixiang Li, Department of Gastroenterology, Qilu Hospital of Shandong University, 107 Wenhuaxi Road, Jinan, Shandong Province, China, 250012, Email: [Lilixiang@sdu.edu.cn](mailto:Lilixiang@sdu.edu.cn)


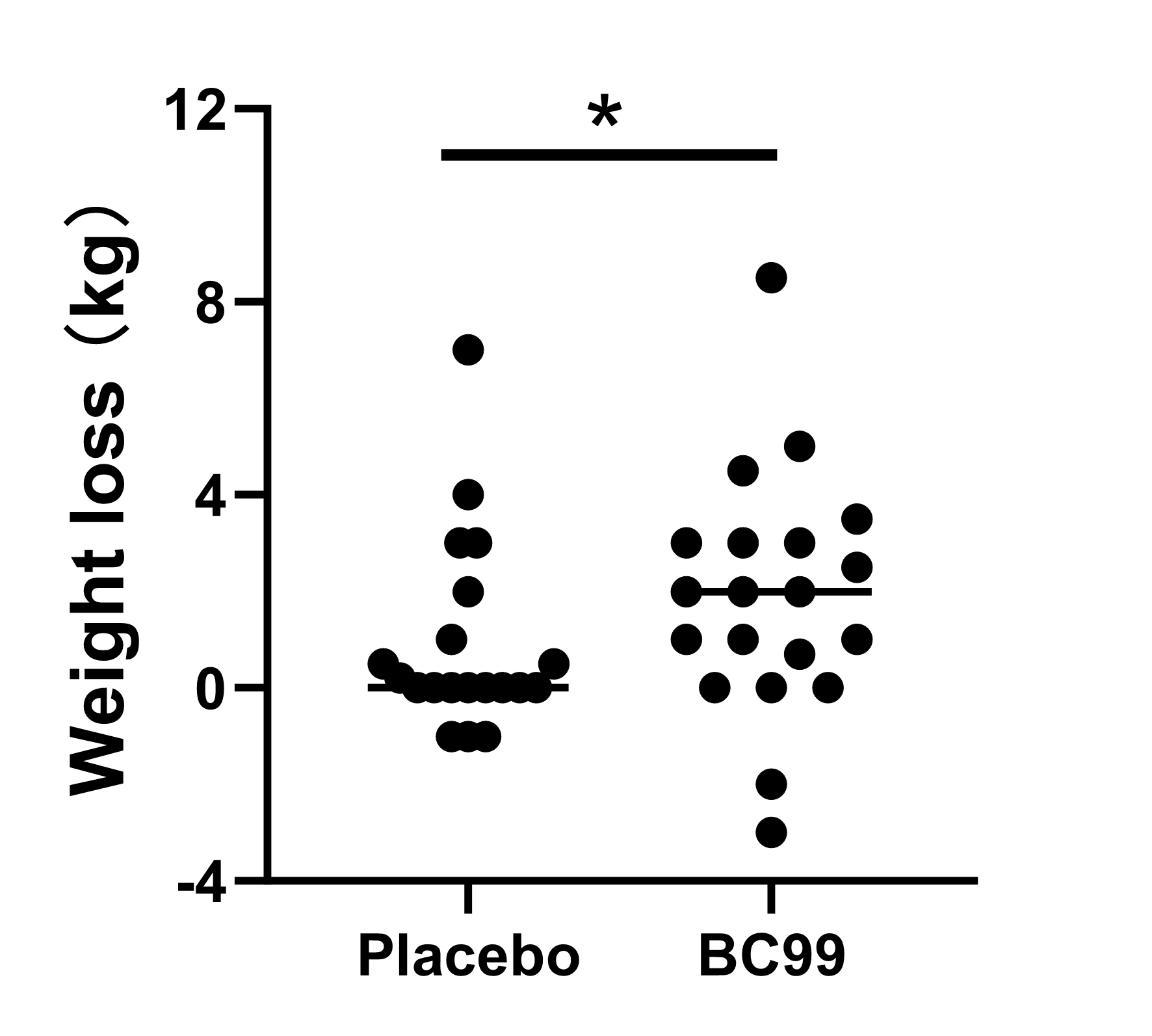


Figure S1. Comparation of weight loss of overweight subjects in probiotic and placebo group at week 8.


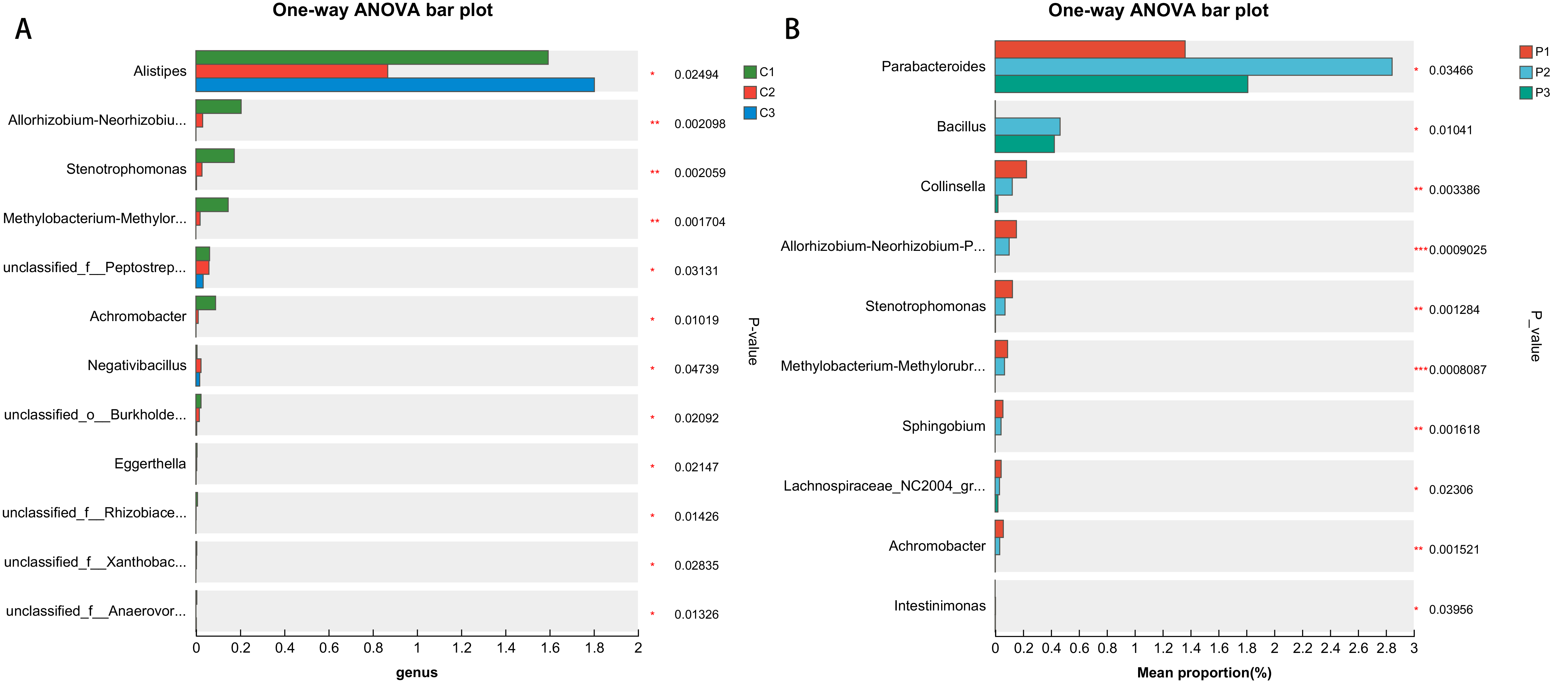


Figure S2. Differences in gut microbiota (genus level) of the probiotic group and the placebo group in week 0, week 4 and week 8. P: probiotic group; C: placebo group; P1/C1: pretreatment; P2/C2: 4 weeks of probiotic intervention; P3/C3: 8 weeks of the probiotic intervention.
